# The rapidly growing landscape of RAS inhibitors: from selective allele blockade to broad inhibition strategies

**DOI:** 10.1002/1878-0261.70149

**Published:** 2025-10-29

**Authors:** Matthias Drosten, Mariano Barbacid

**Affiliations:** ^1^ Centro de Investigación del Cáncer (CIC) Consejo Superior de Investigaciones Científicas (CSIC)‐Universidad de Salamanca (USAL) Salamanca Spain; ^2^ Instituto de Biología Molecular y Celular del Cáncer (IBMCC) CSIC‐USAL Salamanca Spain; ^3^ Centro de Investigación Biomédica en Red de Cáncer (CIBERONC) Instituto de Salud Carlos III Madrid Spain; ^4^ Tumor Biology Program Centro Nacional de Investigaciones Oncológicas (CNIO) Madrid Spain

**Keywords:** combination therapies, drug resistance, KRAS, RAS inhibitors

## Abstract

RAS oncoproteins have been considered undruggable for decades. However, recent advances have led to the development of a large variety of RAS inhibitors. While a multitude of drugs is currently under clinical evaluation, some of them have already been approved in specific contexts, leading to a continuous improvement of available therapeutic options for patients with *RAS*‐mutant cancers. In this Viewpoint, we discuss different classes of RAS inhibitors, with emphasis on those that are currently tested in the clinic including allele‐specific KRAS, panKRAS, and panRAS inhibitors. We also address determinants of response to RAS inhibition such as the tumor context or potential resistance mechanisms. Finally, we provide an outlook on the future of RAS targeting, which is likely to involve combination therapies. The rapid approval of several RAS inhibitors reflects the urgency to develop novel therapeutic strategies to treat *RAS*‐mutant cancers.

## Graphical abstract

RAS‐targeted therapies have become a clinical reality. Although promising results in patients have been achieved, there are still challenges ahead such as coping with drug resistance. Here we summarize selected RAS inhibitors targeting either individual KRAS mutant isoforms (KRAS^G12C^, KRAS^G12D^, KRAS^G12V^) or exhibiting a wider inhibitory spectrum (panKRAS and panRAS), classified by RAS nucleotide states and their stage of clinical development.

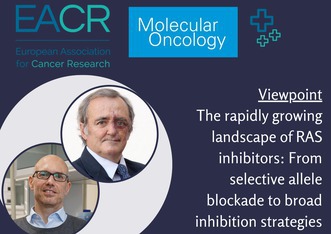

**Abbreviations**
FDA: U.S. Food and Drug Administration
GAP: GTPase‐activating protein
GDP: guanosine diphosphate
GTP: guanosine triphosphate
MAPK: mitogen‐activated protein kinase
NMPA: National Medical Products Administration
PI3K: phosphatidyl inositol 3‐kinase

RAS proteins are small GTPases that cycle between an inactive, GDP‐bound (OFF) state and an active, GTP‐bound (ON) state, in which they transmit signals from extracellular receptors to promote cell proliferation or block apoptosis, among other functions. *KRAS*, the most frequently mutated *RAS* gene in human cancer, is altered in approximately 30% of lung adenocarcinomas (LUAD), 90% of pancreatic ductal adenocarcinomas (PDAC), and 50% of colorectal tumors (CRC). These mutations, mostly affecting codons 12, 13, or 61, impair to varying degrees the ability of KRAS to hydrolyze GTP, leading to a net accumulation of GTP‐bound, active KRAS. Thus, there has been a constant interest in developing selective therapeutic strategies to target KRAS oncoproteins ever since their discovery over 40 years ago [1]. Unfortunately, these early attempts were unsuccessful due to challenges in identifying suitable drug binding pockets, ultimately attributing the notion of undruggability to KRAS oncoproteins. Similarly, strategies to block downstream effector pathways such as the components of the MAPK or PI3K signaling pathways did not produce significant benefits for patients with *KRAS*‐mutant tumors [2].

Fortunately, this situation finally changed when Shokat and colleagues identified compounds that target the highly reactive cysteine residue of KRAS^G12C^, the mutant isoform most frequently present in LUAD. These inhibitors bind a previously unrecognized allosteric pocket beneath the switch II region of KRAS implicated in effector binding [3]. Yet, this pocket is only accessible in its OFF conformation. Extensive follow‐up work by multiple laboratories and pharmaceutical companies led to a gradual improvement of these compounds and culminated in the discovery, preliminary clinical validation, and accelerated FDA approval of two covalent KRAS^G12C^ OFF‐state inhibitors, sotorasib and adagrasib [4] (Fig. 1). This body of work also confirmed the concept that mutant KRAS isoforms, and especially KRAS^G12C^, can still hydrolyze GTP, which is promoted by atypical GAPs such as RGS3 [5].

**Fig. 1:**
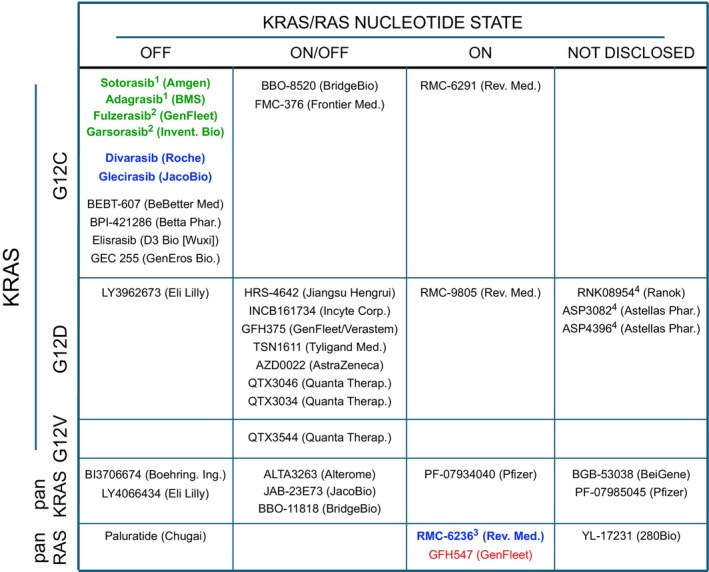
Clinical development of selected KRAS/RAS inhibitors. KRAS^G12C^, KRAS^G12D^, KRAS^G12V^, panKRAS, and panRAS inhibitors are indicated based on the nucleotide state (on/off) of the targeted RAS oncoproteins and the stage of clinical development. Red, preclinical. Black, phase 1/2. Blue, phase 3. Green, approved. Not all inhibitors currently tested in clinical trials are shown. ^1^Approved by the FDA. ^2^Approved by the NMPA of China. ^3^FDA breakthrough therapy designation for PDAC. ^4^PROTACs.

Despite this revolutionary breakthrough, the clinical performance of sotorasib turned out to be less exciting than expected. Data from the CodeBreak 200 phase 3 clinical trial only revealed a minor extension of progression‐free survival (PFS) in pretreated LUAD patients when sotorasib was compared with docetaxel, the standard chemotherapy treatment for these tumors. Moreover, no differences in overall survival (OS) were observed [6]. More recent data from the KRYSTAL‐12 phase 3 trial comparing adagrasib with docetaxel exposed similar findings on PFS while the OS data are yet to be reported [7]. These results can be attributed, at least in part, to the rapid onset of adaptive resistance, which appears to be mediated via a plethora of mechanisms including *KRAS* ^G12C^ amplifications, novel *KRAS* mutations in *cis* or *trans*, mutations in other *RAS* paralogs, upstream receptors or members of downstream signaling pathways [4, 8]. Yet, there is still no information regarding the potential therapeutic benefit of co‐targeting these secondary mutations in patients who developed resistance. Moreover, nongenetic mechanisms such as adeno‐to‐squamous transition, alveolar differentiation or transcriptional adaptation have also been observed [4, 8]. Thus, novel clinical trials are currently in progress to assess potential combinations with these KRAS^G12C^ inhibitors. Combinations with immune checkpoint inhibitors (ICIs) were considered highly promising based on preclinical data. Recent clinical data revealed unexpected hepatotoxicity when combining sotorasib with ICIs, a complication that does not seem to develop when combining adagrasib and ICIs [4, 9, 10]. Other promising combinations might include those that contribute to reducing the proportion of active GTP‐bound KRAS via inhibition of upstream regulators such as SHP2 or SOS1 [9].

KRAS^G12C^ inhibitors were also used in clinical trials with PDAC and CRC patients, although this mutation is much less frequent in these tumors than in LUAD. Preliminary results from a phase 1/2 trial with sotorasib in PDAC patients revealed significant response rates, although PFS (4 months) and OS (7 months) remained unacceptably low [11]. In addition, neither sotorasib nor adagrasib was particularly effective in CRC patients. However, the combination of KRAS^G12C^ inhibitors with EGFR blockers such as cetuximab increased response rates and extended patient survival beyond the effect of KRAS inhibitors in monotherapy, ultimately granting accelerated FDA approval of adagrasib plus cetuximab for *KRAS* ^G12C^‐mutated CRC [12]. Nevertheless, further studies are necessary to substantiate these findings.

A plethora of additional KRAS^G12C^ OFF‐state inhibitors has subsequently been developed, which are currently under clinical evaluation (Fig. 1). Two of these inhibitors, fulzerasib (GFH925) and garsorasib (D‐1553), were recently approved by the NMPA in China, and multiple other drugs have already advanced to phase 3 trials. Yet, resistance is likely to continue to be a major constraint for patient survival, suggesting that combinations might also be key for these more recently developed inhibitors. In addition, novel classes of KRAS^G12C^ inhibitors have been developed that may overcome the limited potential of OFF‐state inhibitors. For instance, BBO‐8520 is a drug capable of covalently binding to the cysteine residue in KRAS^G12C^ regardless of its nucleotide status, thus expanding the range of targetable KRAS species by including GTP‐bound molecules. A second newly developed inhibitor, FMC‐376, is also capable of targeting KRAS^G12C^ in its ON and OFF states (Fig. 1). A distinct class of inhibitors is also now under clinical evaluation that acts as a molecular glue, creating a covalent trimeric complex with KRAS and the chaperone cyclophilin A [8, 13]. These drugs selectively target the ON state and prevent effector binding via sterical hindrance. One of these drugs, RMC‐6291 (elironrasib), has also shown promising preclinical activity and is currently tested in a phase 1/2 trial.

The success of KRAS^G12C^ inhibitors has fueled the development of other allele‐specific inhibitors. Indeed, targeting the most frequent mutant KRAS isoform, KRAS^G12D^, became a priority for drug development efforts. The first highly selective compound, MRTX1133, which non‐covalently inhibited KRAS^G12D^ making use of the same switch II pocket, showed encouraging preclinical activities but its clinical development was recently discontinued due to unfavorable pharmacological properties. Nevertheless, a multitude of novel KRAS^G12D^ inhibitors with more favorable properties have recently emerged. Most of these inhibitors target KRAS^G12D^ independently of its nucleotide state, while some show a more preferential activity toward the inactive variant (Fig. 1). Interestingly, among those inhibitors currently under clinical evaluation are KRAS^G12D^‐selective PROteolysis TArgeting Chimeras (PROTACs) which selectively target this oncoprotein for proteasomal degradation [14]. While technically more challenging to develop, these PROTACs combine several advantages over classical inhibitors that could result in enhanced activity. Yet, their clinical efficacy remains to be revealed. Finally, the KRAS^G12D^ molecular glue RMC‐9805 (zoldonrasib) selective for the active state is also currently tested in the clinic.

While some of these allele‐specific inhibitors have indeed shown encouraging activities in patients, they may come with the caveat of being ineffective against novel *KRAS* mutations in *cis* or *trans* that emerge in resistant tumors. To this end, panKRAS inhibitors (also known as KRAS multi‐inhibitors) that target both wild‐type KRAS as well as a broad range of mutations have been developed (Fig. 1). Although no clinical data have been reported yet, panKRAS inhibitors may indeed possess superior efficacy over allele‐specific inhibitors. However, it is currently unclear whether they may possess a less favorable safety profile, although *Kras* was shown to be largely dispensable for the survival of adult mice [15]. In addition, panKRAS inhibitors may also be an option for patients who have developed resistance to allele‐specific inhibitors.

Analysis of tumor cells from patients who developed resistance also revealed novel mutations in other *RAS* paralogs such as *NRAS* or *HRAS* as well as mutations in upstream activators including EGFR that can cause reactivation of wild‐type RAS paralogs [4, 8, 9, 10]. Thus, the development of panRAS inhibitors could offer even broader clinical activities further reducing the potential to develop resistance. Indeed, the recent panRAS molecular glue ON‐state inhibitor RMC‐6236 (daraxonrasib) has shown surprising clinical efficacy and a manageable safety and tolerability profile [16], with multiple other panRAS inhibitors currently under preclinical and clinical evaluation (Fig. 1). Despite the essential requirement for RAS signaling in adult mice [15], there is a wide therapeutic window that allows the safe use of panRAS inhibitors in the clinic [16]. Indeed, data from a recent clinical trial with daraxonrasib have shown promising response rates and a considerably increased patient survival as second‐ or third‐line treatment. These results have led to a recent FDA breakthrough therapy designation for this compound in PDAC patients with *KRAS* mutations in codon 12 [17]. Interestingly, a recent molecular analysis has shown that this inhibitor is characterized by a dual mechanism of action. In addition to sterical hindrance of effector binding, it also stimulates GTP hydrolysis of codon 12 mutant isoforms leading to more pronounced and durable inhibition [18]. Nevertheless, preclinical studies with this inhibitor or its related tool compound RMC‐7977 have indicated that this type of inhibitor is not exempt from developing resistance, an observation that was recently confirmed in patients treated with daraxonrasib [19].

Hence, combining these panRAS (ON) inhibitors with other drugs is likely to further increase their efficacy. Indeed, two recent preclinical studies have shown that combining RMC‐7977 with ICIs [20] or the CDK4/6 inhibitor palbociclib [21] conferred deeper and more durable regressions in experimental models of PDAC. More recent data using a genetic approach involving ablation of three independent KRAS signaling nodes have led to complete regression of KRAS^G12V^‐driven experimental PDAC and prevented the onset of tumor resistance [22]. Translation of these results to a pharmacological scenario has illustrated that combining daraxonrasib with afatinib, an irreversible EGFR/HER2 tyrosine kinase inhibitor, and SD36, a selective STAT3 PROTAC, also led to robust tumor regression without evidence of tumor resistance [22]. Yet, the use of this triple therapy in the clinic will require improved tolerance. Identification of better‐tolerated EGFR/HER2 and STAT3 inhibitors should make this therapeutic strategy suitable for the treatment of PDAC patients.

Overall, the landscape of clinical KRAS/RAS inhibitors is rapidly evolving, which in the long term can only be beneficial for patients suffering from *RAS* mutant tumors. Yet, the field is still at its beginning, and a plethora of questions remain to be resolved, such as which inhibitor will be most efficient for which patients and in which combination. However, given the long trajectory of clinical failures for patients with *RAS* mutations, a new era of optimism has finally arrived.

## Conflict of interest

MB is a co‐inventor of a European Patent Application No. 25382815.6. MD has no conflict of interest to declare.

## Author contributions

MD and MB conceptualized the Viewpoint, conducted the literature review, and wrote the manuscript.
